# Amphiphilic Quantum Dots with Asymmetric, Mixed Polymer Brush Layers: From Single Core-Shell Nanoparticles to Salt-Induced Vesicle Formation

**DOI:** 10.3390/polym10030327

**Published:** 2018-03-16

**Authors:** Brian R. Coleman, Matthew G. Moffitt

**Affiliations:** Department of Chemistry, University of Victoria, P.O. Box 3065, Victoria, BC V8W 3V6, Canada; Brian_Coleman33@hotmail.com

**Keywords:** quantum dots, self-assembly, mixed polymer brush nanoparticles, vesicles, core-shell nanoparticles, fluorescence

## Abstract

A mixed micelle approach is used to produce amphiphilic brush nanoparticles (ABNPs) with cadmium sulfide quantum dot (QD) cores and surface layers of densely grafted (σ = ~1 chain/nm^2^) and asymmetric (*f*_PS_ = 0.9) mixed polymer brushes that contain hydrophobic polystyrene (PS) and hydrophilic poly(methyl methacrylate) (PMAA) chains (PS/PMAA-CdS). In aqueous media, the mixed brushes undergo conformational rearrangements that depend strongly on prior salt addition, giving rise to one of two pathways to fluorescent and morphologically disparate QD-polymer colloids. (A) In the absence of salt, centrosymmetric condensation of PS chains forms individual core-shell QD-polymer colloids. (B) In the presence of salt, non-centrosymmetric condensation of PS chains forms Janus particles, which trigger anisotropic interactions and amphiphilic self-assembly into the QD-polymer vesicles. To our knowledge, this is the first example of an ABNP building block that can form either discrete core-shell colloids or self-assembled superstructures in water depending on simple changes to the chemical conditions (i.e., salt addition). Such dramatic and finely tuned morphological variation could inform numerous applications in sensing, biolabeling, photonics, and nanomedicine.

## 1. Introduction

Colloidal quantum dots (QDs) have become a cornerstone of modern materials research, due to their compelling size- and shape-dependent optical properties [1,2,3,4,5]. A key challenge for a wide range of future applications in photonics, bioimaging, and nanomedicine is the controlled spatial organization of QDs into hierarchical composite assemblies with targeted and complex functionality [1]. Several groups have met this challenge through the introduction of block copolymers or mixed polymer brush layers at the surfaces of QDs or other inorganic nanoparticles [6,7,8,9,10,11,12,13,14,15]. Chemically dissimilar polymer segments in the polymer layers are either specifically patterned or else undergo localized segregation on the nanoparticle surfaces, triggering anisotropic interactions in selective solvents and amphiphilic self-assembly into a variety of multiscale nanoparticle superstructures. 

In our group, we have previously used the triblock copolymer polystyrene-*block*-poly(acrylic acid)-*block*-poly(methyl methacrylate) (PS-*b*-PAA-*b*-PMMA) to form inverse micelles that were then used as templates to grow individual cadmium sulfide (CdS) QDs in each micelle core [16]. Subsequent crosslinking of the micelle cores and hydrolysis of PMMA chains to poly(methacrylic acid) (PMAA) formed amphiphilic brush nanoparticles (ABNPs) with QD cores and mixed brush layers consisting of equal numbers of hydrophobic PS and hydrophilic, ionizable PMAA chains [17]. The ABNPs underwent self-assembly in tetrahydrofuran (THF)/water mixtures to generate spherical supermicelles, vesicles, and segmented wormlike aggregates depending on the chemical conditions, including salt content [17].

Along with self-assembled QD-polymer superstructures, individual aqueous core-shell nanoparticles that consist of an inorganic core, a condensed hydrophobic polymer shell, and hydrophilic stabilizing ligands are of specific interest for various applications in biolabeling, sensing, and photonics [18]. For instance, core-shell colloids have been produced by the encapsulation of QDs bearing hydrophobic surface ligands within the cores of block copolymer micelles in aqueous media [19]. To our knowledge, a method that can form either individual core-shell colloids or self-assembled superstructures depending on chemical conditions via a common QD-polymer building block has not been previously demonstrated.

**Figure 1:**
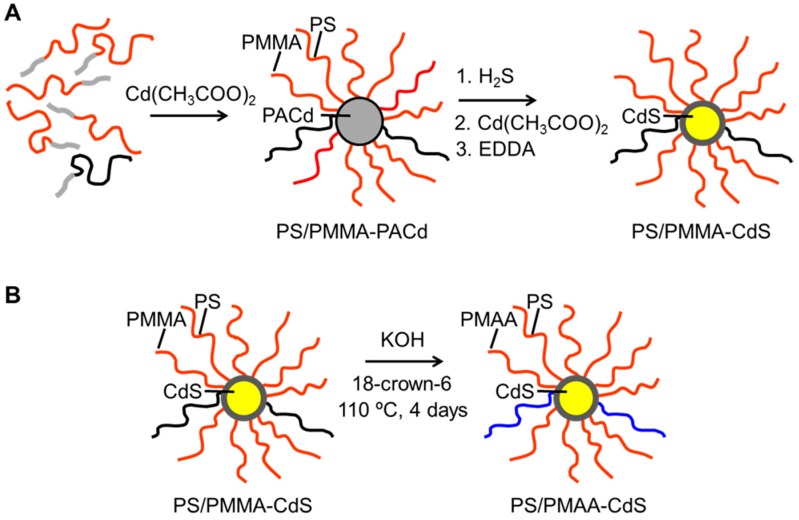
Schematic showing preparation routes (**A**) from block copolymer blend to hydrophobic mixed brush nanoparticle PS/PMMA-CdS and (**B**) from PS/PMMA-CdS to amphiphilic brish nanoparticle PS/PMAA-CdS.

In this paper, we use a mixed micelle approach to produce ABNPs with CdS QD cores and a dense PS/PMAA mixed brush layer of highly asymmetric composition (mole fraction of PS chains, *f*_PS_ = 0.9). Specifically, we first form reverse micelles from an asymmetric blend of PS-*b*-PAA and PMMA-*b*-PAA diblock copolymers in organic solvent followed by templated CdS growth and crosslinking in the micelle cores (Figure 1A). Next, PMMA hydrolysis to PMAA results in ABNPs with a mixed polymer brush layer of 90 mol·% hydrophobic PS chains and 10 mol·% hydrophilic PMAA chains (Figure 1B). Slow water addition to ABNPs dispersed in THF is found to induce one of two conformational rearrangements: (A) in the absence of salt, centrosymmetric condensation of PS chains forms individual core-shell QD-polymer colloids; or (B) in the presence of salt, non-centrosymmetric condensation of PS chains forms Janus particles with anisotropic interactions, which lead to self-assembly into QD-polymer vesicles. The interesting, salt-dependent switching between these two pathways is the combined result of the asymmetric composition and dense brush structure formed from the *f*_PS_ = 0.9 blend.

## 2. Materials and Methods 

### 2.1. Materials

Two constituent block copolymer starting materials were purchased from Polymer Source Inc. (Montreal, QC, Canada): polystyrene-*block*-poly(*tert*-butyl acrylate) (PS(225)-*b*-P*t*BA(22), *M*_w_/*M*_n_ = 1.06) and poly(methyl methacrylate)-*block*-poly(tert-butyl acrylate) (PMMA(225)-*b*-PtBA(21), *M*_w_/*M*_n_ = 1.08), in which the numbers in brackets indicate the number-average degrees of polymerization for each block.

### 2.2. Hydrolysis of PS-b-PtBA to PS-b-PAA and Selective Hydrolysis of PMMA-b-PtBA to PMMA-b-PAA

Each of the two copolymers were hydrolyzed to the poly(acrylic acid) (PAA) form in order to create inverse micelles with an ionomer core based on the PAA blocks. PS-*b*-P*t*BA was hydrolyzed to PS-*b*-PAA, and PMMA-*b*-PtBA was hydrolyzed to PMMA-*b*-PAA using the following method. The *tert*-butyl acrylate form of each block copolymer was dried overnight at 70 °C under vacuum then dissolved in toluene to prepare a 2 wt % solution. The clear, colourless solution was stirred overnight to insure complete dispersal of the copolymer. The solution was then refluxed at 115 °C overnight with *para*-toluenesulfonic acid (5 mol·% relative to the *tert*-butyl acrylate content). The dark brown solution was then concentrated to half of its initial volume via rotary evaporation and precipitated into isopropanol cooled by an ice/water bath to 0 °C. The resulting white powder was then recovered by vacuum filtration, washed further with 3 × 5 mL cold isopropanol, and dried in a vacuum oven at 70 °C for two days. Complete hydrolysis of PS-*b*-P*t*BA to PS-*b*-PAA was confirmed by FTIR, and complete selective hydrolysis of PMMA-*b*-P*t*BA to PMMA-*b*-PAA, without significant hydrolysis of the PMMA block, was confirmed by ^1^H NMR.

### 2.3. Preparation of Inverse Mixed Micelles of PS-b-PAA and PMMA-b-PAA (PS/PMMA-PACd)

The PS(225)-*b*-PAA(22) and PMMA(225)-*b*-PAA(21) components were each dissolved in a 90/10 (*v/v*) benzene/methanol mixture to prepare two separate 2 wt % solutions, which were stirred overnight to equilibrate. The targeted asymmetric blend composition is designated by the number fractions of polystyrene chains (*f*_PS_ = 0.9). The two stock solutions of PS(225)-*b*-PAA(22) and PMMA(225)-*b*-PAA(21) were blended gravimetrically with the appropriate blend ratio. The blend solution was allowed to stir for two hours, then cadmium acetate dihydrate (Aldrich, 3 molar equivalents per mole acrylic acid repeat units) in 0.25 M methanolic solution was added in a continuous stream under constant stirring to neutralize the acrylic acid blocks and trigger mixed micelle formation. The resulting blue-tinged dispersion was stirred overnight then freeze-dried to remove the solvent, producing a white powder that was washed repeatedly with methanol, then dried overnight in a vacuum oven at 70 °C. GPC analysis of the mixed micelle sample in THF showed a single chain fraction <10 wt %. The resulting sample was designated PS/PMMA-PACd. 

### 2.4. Preparation of PS/PMMA Mixed Polymer Brush-Stabilized CdS Nanoparticles (PS/PMMA-CdS) 

The PS/PMMA-PACd white powder was placed in an atmosphere of 100% humidity at 70 °C for one week. The powder was then exposed to wet hydrogen sulfide (H_2_S) for 10 h, resulting in colour changes from white to yellow, and then stored under active vacuum overnight to remove excess H_2_S gas. Treatment with H_2_S resulted in the growth of a single CdS nanoparticle in each mixed micelle core, but also protonation of the PAA blocks, which destabilizes the surrounding micelles. Therefore, the mixed brush layers surrounding the CdS cores were crosslinked in the following manner. The yellow powder was first ground into fine powder and then dispersed in THF to 2 wt %. Immediately upon dispersion, cadmium acetate dihydrate (0.5 molar equivalents per mole acrylic acid repeat units) in 0.25 M methanolic solution were added, and the dispersion was allowed to stir overnight. Next, a 1 wt % solution of *N*-ethyl-*N*’-(3-dimethylaminopropyl)-carbodiimide methiodide (EDC) activator in water (0.5 molar equivalents per mole acrylic acid repeat units) was added and allowed to stir for 2 h. This was followed by the addition of a 1 wt % solution of 2,2’-(ethylenedioxy)bis(ethylamine) (EDDA) in water (0.25 molar equivalents per mole acrylic acid repeat units). After overnight stirring, the dispersion was concentrated by rotary evaporation then precipitated into cold methanol. The resulting pale yellow solid was filtered and dried under vacuum for 2 days at 70 °C. The resulting sample of PS/PMMA mixed brush-stabilized CdS nanoparticles was designated PS/PMMA-CdS. 

### 2.5. Hydrolysis of PMMA Chains to Form Amphiphilic PS/PMAA Mixed Polymer Brush-Stabilized CdS Nanoparticles (PS/PMAA-CdS) 

The hydrophobic PMMA chains in the PS/PMMA-CdS nanoparticles were hydrolyzed to PMAA to produce hydrophobic/hydrophilic brush nanoparticles of variable composition. First, the yellow powder PS/PMMA-CdS was dispersed to 10 wt % in 1,4-dioxane with KOH (2 molar equivalents per mole methyl methacrylate repeat units) and 18-crown-6 (0.2 molar equivalents per mole methyl methacrylate repeat units). The dispersion was placed under argon in a sealed high-pressure Schlenk tube, and the reaction was heated to 110 °C for 4 days. After 4 days, the reaction mixture was concentrated and precipitated into cold 0.2 M acetic acid. The powders were filtered and dried under active vacuum for 3 days at 70 °C. The resulting sample of amphiphilic nanoparticles was designated PS/PMAA-CdS. 

### 2.6. Investigation of PS/PMAA-CdS Self-Assembly Behaviour in THF/Water Mixtures 

PS/PMAA-CdS was dispersed in THF (≥99.9% HPLC grade, Sigma Aldrich (St. Louis, MO, USA), [H_2_O] < 0.02%) to initial concentrations of *c_o_* = 0.25, 0.50 or 0.75 wt %. For some experiments, 3.0 M NaCl reagent grade (EMD, Darmstadt, Germany) solutions in deionized water were added prior to self-assembly such that the ratio of NaCl to methacrylic acid repeat units was *R*_NaCl_ = 1.5 or *R*_NaCl_ = 3.0 (the added water in the salt solutions was well below the critical water content in all cases). The dispersions were set to a constant, rapid stirring rate, as deionized water was added dropwise at a rate of 10 µL/10 s. The point at which the solution became turbid was noted as the critical water concentration (CWC) for self-assembly. Dropwise water addition was continued up to 75 wt %. The dispersion was then poured immediately into 10 mL of deionized water, followed by dialysis against 500 mL of deionized water for 5 days to remove THF (dialysis tubing consisted of regenerated cellulose with a 50,000 molecular weight cut-off, Spectrum Labs.); during dialysis, water was changed every hour for the first 4 hours then every 12 h for the remaining period. All dispersions were covered in aluminum foil to protect against decomposition from light sources. The room temperature during self-assembly was maintained at 22 ± 1 °C. Following dialysis, all aqueous colloids were stored in a dark drawer. 

### 2.7. Gel Permeation Chromatography (GPC)

GPC measurements were performed using a Viscotek Model 302 (Malvern Panalytical, Malvern, Worcestershire, UK) liquid chromatography system equipped with refractive index (RI), low-angle light scattering (LALS, θ = 7°), right-angle light scattering (RALS, θ = 90°), and UV detectors. THF was used as the eluent at a flow rate of 1 mL/min, and the column temperature was set to 35 °C. All polymer dispersions were filtered through membrane filters with a nominal pore size of 0.45 μm before injection into the GPC column. The data were collected and analyzed on a Dell Dimension 2300 computer (Dell, Round Rock, TX, USA) with appropriate GPC software from Viscotek. Three styrene-divinyl benzene columns (Tosoh Biosciences, San Francisco, CA, USA) were connected in series: a TSKgel G3000HHR column, followed by a TSKgel GM-HHR-M column, followed by a second TSKgel G3000HHR column. 

### 2.8. ^1^H NMR Analysis 

^1^H NMR spectra of PS/PMMA-PACd mixed micelles in chloroform-d were used to determine relative PS and PMMA chain fractions. Spectra were recorded using a Bruker AC 300 MHz spectrometer (Bruker, Billerica, MA, USA).

### 2.9. UV–Vis Absorption Measurements 

Absorption spectra were recorded on a PerkinElmer UV–Vis-NIR (PerkinElmer, Waltham, MA, USA) with a three detector module consisting of a photomultiplier tube (PMT) for the UV–Vis range, an indium gallium arsenide (InGaAs) detector, and a lead sulfide (PbS) detector for the near infra-red (NIR) range. All samples were dispersed to ~1 mg/mL is spectroscopic grade THF, toluene, acetone, and chloroform. A background of pure solvent was subtracted from each spectrum. 

### 2.10. Static and Dynamic Light Scattering Measurements

Static light scattering (SLS) and dynamic light scattering (DLS) experiments were carried out on a Brookhaven Instruments photon correlation spectrometer (Holtsville, NY, USA) equipped with a BI-200SM goniometer, a BI-9000AT digital autocorrelator, and a Melles Griot He-Ne Laser (632.8 nm) with a maximum power output of 75 mW. To ensure the accuracy of both SLS and DLS measurements, great care was taken to eliminate dust from the samples. Spectroscopic grade THF was filtered through two membrane filters with 0.20 μm nominal pore size connected in series. Stock solutions (ca. 5 mg/mL) of PS/PMMA-CdS colloids in THF were prepared the night before SLS or DLS measurements to ensure equilibration and then filtered through two membrane filters with 0.45 μm nominal pore size connected in series to remove dust. All scintillation vials were thoroughly cleaned with filtered solvent. All measurements were carried out at 23 °C. 

SLS measurements of PS/PMMA-CdS in THF were carried out in a concentration range from 0.05 to 1 mg/mL. For each concentration, 12 different angles of detection from 30° to 120° were measured. Ten repeat measurements of scattered light intensity were taken at each angle and concentration to obtain Zimm plots. Reported values of *M_w_* and root-mean-square *z*-average radii of gyration, *r_g_*, were determined from the average results of at least two separate Zimm plots obtained from separate dilutions of the same stock solution. 

Differential refractive index values, d*_n_*/d*_c_*, for the three different PS/PMMA-CdS samples dispersed in THF were required for Zimm plot analysis of static light scattering results. These were determined using a BI-DNDC Differential Refractometer that was calibrated using a known standard of potassium chloride (KCl) in water. Stock solutions of PS/PMMA-CdS of ca. 10 mg/mL were diluted to obtain 5 concentrations from 1 to 5 mg/mL for d*_n_*/d*_c_*determination. Three repeat measurements of d*_n_*/d*_c_* were obtained for PS/PMMA-CdS in THF. 

DLS measurements of PS/PMMA-CdS in THF were conducted at a single scattering angle of 90° and at three different concentrations in the range of ~0.2–~0.8 mg·mL^−1^. For each concentration, three repeat measurements of the autocorrelation function were obtained. The single-particle hydrodynamic diameter, *d*_h,0_, of PS/PMMA-CdS in THF was determined by extrapolation of effective hydrodynamic diameters measured at each concentration to infinite dilution. 

### 2.11. Laser Scanning Confocal Fluorescence Microscopy (LSCFM)

Laser scanning confocal fluorescence microscopy measurements were done on a Zeiss LSM 410 (Carl Zeiss AG, Oberkochen, Germany) equipped with an Ar/Kr laser. A Zeiss Plane-Aprochromat 63x (Carl Zeiss AG, Oberkochen, Germany) oil immersion objective was used. All films were excited at ~488 nm, using a band-pass 485 ± 20 nm line selection filter and a FT 510 dichroic beam splitter. A long-pass 515 emission filter was used such that only light above 515 nm reached the PMT. A pinhole diameter of 1.31 Airy Units was used for all measurements, resulting in an optical section thickness of 0.75 μm FWHM. Slides were prepared by placing ~1 mL aqueous PS/PMAA-CdS assemblies between a glass slide and a coverslip. 

### 2.12. Transmission Electron Microscopy 

TEM was performed on a JEOL JEM-1400 electron microscope (JEOL Ltd., Tokyo, Japan), operating at an electron accelerating voltage of 80 kV. For imaging non-assembled PS/PMAA-CdS, two drops of 1 wt % dispersion in THF were deposited on a copper grid (300 mesh) coated with an amorphous carbon film and blotting excess solution after 15 s; the grids were then dried at room temperature for 2 h before imaging. Particle size measurements and statistical analysis of non-assembled CdS nanoparticles in THF were performed on the ImageJ software from Softonic (Barcelona, Spain). To measure average CdS nanoparticle sizes from TEM, three images were taken from three different randomly selected regions of each grid, from which a minimum of 200 total particles were measured. For TEM imaging of PS/PMAA-CdS core-shell nanoparticles and vesicle assemblies in aqueous dispersions, the original dialyzed dispersions were deposited without dilution on a carbon-coated 300-mesh copper grid with blotting of the excess solution after 2 min; the grids were then dried at room temperature overnight before imaging. Average PS core sizes and standard deviations were compiled from measurement of at least 50 particles.

## 3. Results and Discussion

### 3.1. Characterization of Inverse Mixed Micelles (PS/PMMA-PACd)

To produce the polymeric mixed brush template for CdS nanoparticle synthesis, co-micellization of an *f*_PS_ = 0.90 blend of PS-*b*-PAA and PMMA-*b*-PAA in benzene/methanol was initiated by addition of cadmium acetate to produce inverse mixed micelles (PS/PMMA-PACd) with insoluble poly(cadmium acrylate) (PACd) cores and solubilized mixed brush coronae (Figure 1A). Upon addition of cadmium acetate to the diblock copolymer blend, the dispersion became blue-tinged and slightly turbid, indicating increased Rayleigh scattering from the newly formed inverse micelles. The solvent was then removed by freeze-drying with the high-*T*_g_ PACd cores while maintaining structural integrity of the micelles in the absence of solvent. 

PS/PMMA-PACd was dispersed in THF, and GPC was used to determine a single chain content of <10 wt % (Figure S1, red chromatogram, Supplementary Materials). The actual composition of the mixed micelle sample was then determined using ^1^H NMR. Figure S2 (Supplementary Materials) shows the NMR spectrum and peak assignments for the mixed micelles. The mole fraction of PS chains relative to the total number of PS and PMMA chains was determined from the spectrum using relative peak integrations of the aromatic protons of PS (6.2–7.2 ppm) and the methyl ester protons (3.6 ppm) of PMMA. In this manner, the PS mole fraction for the blend was determined to be 0.89, from which we designate the composition of PS/PMMA-PACd to be *f*_PS_ = 0.9. Since the mixed micelles maintain structural integrity in subsequent synthesis steps, the same *f*_PS_ value also refers to the PS/PMMA-CdS and PS/PMAA-CdS samples described in subsequent sections.

### 3.2. Characterization of Hydrophobic Mixed Brush Quantum Dots (PS/PMMA-CdS)

The mixed micelles PS/PMMA-PACd were next used as templates to grow a single CdS QD in each PACd core (Figure 1A). Treatment with H_2_S leads to CdS precipitation but also protonates PAA chains, making the micelles less stable. Therefore, the PAA blocks were crosslinked by first treating with cadmium acetate, followed by addition of EDDA, to obtain CdS QDs surrounded by a mixed brush layer sufficiently robust for the rigorous conditions of PMMA hydrolysis. The resulting yellow powder was designated PS/PMMA-CdS. Comparison of GPC traces in THF of PS/PMMA-PACd (Figure S1, red chromatogram, Supplementary Materials) and PS/PMMA-CdS (Figure S1, black chromatogram, Supplementary Materials) indicated a general relative shift in the nanoparticle peak to higher elution volumes, although without an increase in the single chain fraction. This suggests that CdS growth and crosslinking decreased the hydrodynamic volume of the nanoparticles, likely due to contraction of the core, while maintaining the structural integrity of surrounding polymer brush.

**Figure 2:**
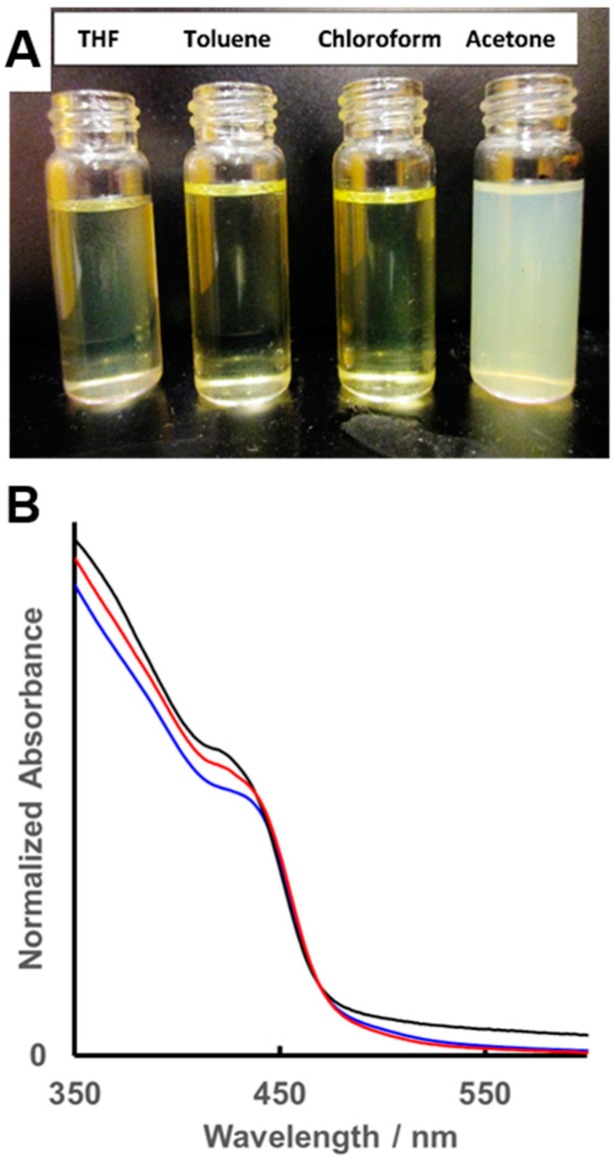
(**A**) Dispersibility tests for PS/PMMA-CdS in solvents of different polarities and (**B**) UV–Vis spectra of PS/PMMA-CdS dispersed in chloroform (blue), toluene (red), and THF (black).

To demonstrate that PS/PMMA-CdS possessed a mixed brush structure, dispersability tests were carried out in four different solvents of varying polarity: THF, toluene, chloroform, and acetone. The first three solvents are good solvents for both PS and PMMA, while acetone is a poor solvent for PS but a good solvent for PMMA. Therefore, if PS-*b*-PAA and PMMA-*b*-PAA were segregated into separate nanoparticles rather than forming mixed brush nanoparticles, then precipitate of PS-*b*-PAA micelles would be expected in acetone. The PS/PMMA-CdS powder yielded clear yellow dispersions in THF, toluene, and chloroform, while forming a turbid but stable (no precipitation) dispersion in acetone (Figure 2A). This confirmed a mixed brush structure in the PS/PMMA-CdS nanoparticles [16]. The clear dispersions of PS/PMMA-CdS in THF, toluene, and chloroform were characterized by UV–Vis absorbance spectroscopy, revealing spectra characteristic of the CdS QD cores (Figure 2B). The spectrum in toluene (red spectrum) shows a blue-shifted absorption threshold (λ_thresh_ = 478 nm) relative to bulk CdS (λ_thresh_ = ~500 nm) and a distinct exciton shoulder (λ_ex_ = ~440 nm) that indicates quantum confinement. In addition, the three spectra in different solvents show strong overlap, suggesting that the polymer layer shields the CdS QDs from the effects of the dielectric properties of the solvent media including Ostwald ripening. 

Light scattering measurements of PS/PMMA-CdS in THF yielded information on the colloidal structure of the mixed brush-coated QDs (Table 1). First, SLS measurements yielded a Zimm plot (Figure S3, Supplementary Materials) from which an apparent weight-average molecular weight (*M*_w_ = 2.5 ± 0.2 × 10^7^ g/mol) and root-mean-square *z*-average radius radii of gyration (*r*_g_ = 123 ± 9 nm) were determined. The measured d*n*/d*c* value in THF, required for *M*_w_ determination, was 0.17, slightly lower than the value for PS in THF (0.185), which is expected since PMMA chains that have a much lower d*n*/d*c* value in THF (0.09) [16]. The PS/PMMA-CdS aggregation numbers, or the total number of PS and PMMA chains in the brush layer, *Z* = 850 ± 70, was determined by dividing *M*_w_ by the unimer molecular weight (2.95 × 10^4^ g/mol). A unimer was assumed to consist of the sum of (1) an “average” copolymer chain of *M_W_* = [0.9 × Mn,PS-b-PAA] + [0.1 × Mn,PMMA-b-PAA], (2) one CdS unit per PAA repeat unit, (3) one Cd^2+^ ion for every four PAA repeat unit, and (4) one EDDA molecule for every four PAA repeat unit. The mean chain surface density was determined by dividing *Z* by the surface area of the core, including the CdS QD and the surrounding crosslinked PACd layer. The volume of the crosslinked PACd layer was approximated using the density of 2 g/mL from the experimentally-determined value for poly(cesium acrylate), yielding a core radius of *r*_core_ = 8.1 ± 0.3 nm [16]. The resulting mean chain surface density is σ = 1.02 ± 0.08 chains/nm^2^. 

Next, DLS measurements of PS/PMMA-CdS in THF provided the single-particle hydrodynamic radius (*r*_h_,_0_ = 29.0 ± 0.2 nm) by extrapolation to infinite dilution of effective hydrodynamic diameters, *d*_h,eff_, determined at three different concentrations (Figure S4A, Supplementary Materials). The hydrodynamic size distribution is determined from CONTIN analysis of DLS data (Figure S4B, Supplementary Materials). Finally, from combined SLS and DLS measurements, we calculated the ratio *r*_g_/*r*_h_,_0_ = 4.2 ± 0.1 for PS/PMMA-CdS in THF. The ratio is higher than would be expected for an isotropic starlike brush (*r*_g_/*r*_h,0_ = 1.1) [16]. _ENREF_3The higher ratio is not due to anisotropic CdS cores, since TEM reveals these to be roughly spherical (Figure S5, Supplementary Materials). Rather, the elevated *r*_g_/*r*_h,0_ ratio is attributed to an anisotropic distribution of scattering centers (PS and PMMA segments) within the asymmetric mixed brushes. 

### 3.3. Characterization of Amphiphilic Mixed Brush Quantum Dots (PS/PMAA-CdS)

**Figure 3:**
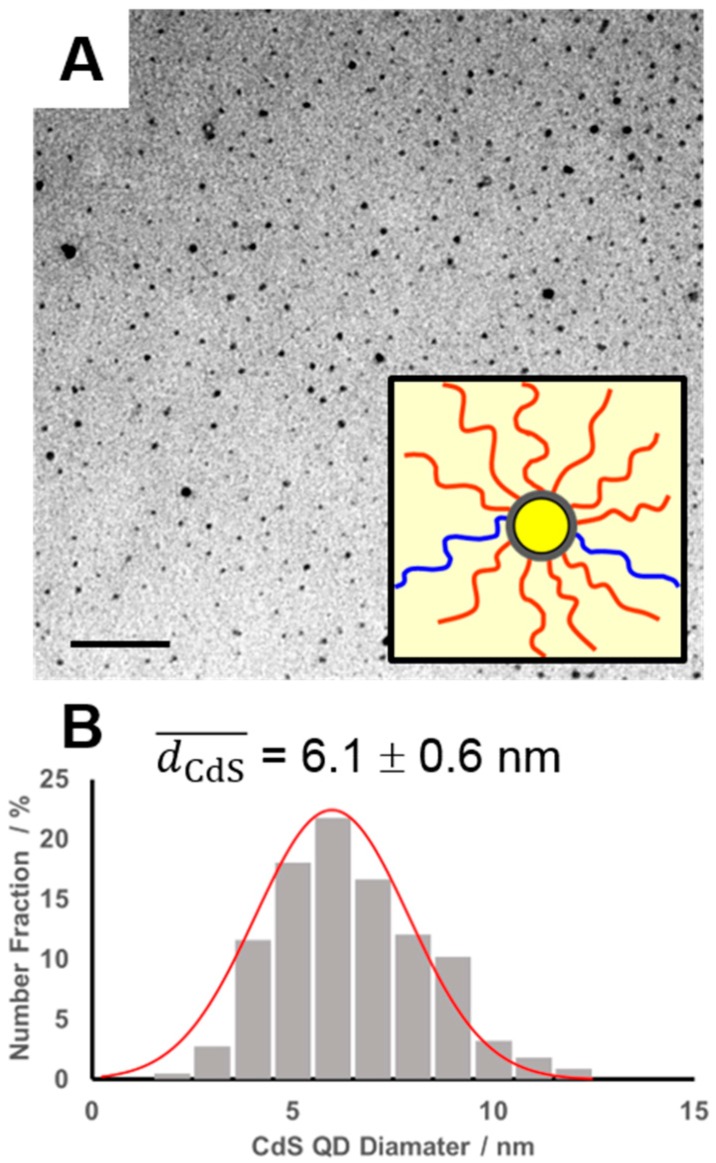
(**A**) TEM showing dark CdS QD cores of THF-cast PS/PMAA-CdS and associated schematic of PS/PMAA-CdS in THF (inset). Scale bar is 100 nm. (**B**) Size distribution and mean diameter of CdS QDs from TEM images. Error on mean value is the standard deviation of three average values determined in three different regions of the TEM grid.

Following characterization of PS/PMMA-CdS, the hydrophobic methyl methacrylate repeat units in the mixed brush were hydrolyzed to hydrophilic methacrylic acid repeat units using KOH catalysis and 18-crown-6 in refluxing dioxane (Figure 1B). The ^1^H NMR spectra, comparing the unhydrolyzed and hydrolyzed forms of PS/PMMA-CdS and PS/PMAA-CdS, respectively, are shown in Supplementary Materials (Figure S6A). The disappearance of the PMMA methoxy peak at 3.60 ppm signifies complete hydrolysis and formation of PMAA in the mixed brush layer. As the hydrolysis conditions were quite robust, it was necessary to determine the structural integrity of the mixed brush nanoparticles by GPC following hydrolysis. GPC traces (Figure S6, Supplementary Materials) before and after hydrolysis (black and blue chromatograms, respectively) show a shift and broadening of the main nanoparticle peak but without a relative increase in the single chain fraction, indicating that the crosslinked polymer layers remained intact during hydrolysis. TEM analysis of PS/PMAA-CdS deposited from THF (Figure 3) revealed roughly spherical CdS QDs that were the same mean size (6.1 ± 0.6 nm) as those present in PS/PMMA-CdS deposited from benzene before hydrolysis (Figure S5, Supplementary Materials), indicating that the QDs also retained structural integrity during the hydrolysis step.

### 3.4. Core-Shell Nanoparticle Formation and Salt-Induced Self-Assembly of Amphiphilic PS/PMAA CdS in Water

Dropwise addition of water to PS/PMAA-CdS dispersions in THF leads to an increase in the solubility of PMAA chains and a decrease in the solubility of PS chains. Eventually, a sudden increase in turbidity indicates microprecipitation by either interparticle or intraparticle association of PS chains. Following continued dropwise water addition to 75 wt % water, the resulting aqueous dispersions were characterized following dialysis against pure water to remove residual THF. 

**Figure 4:**
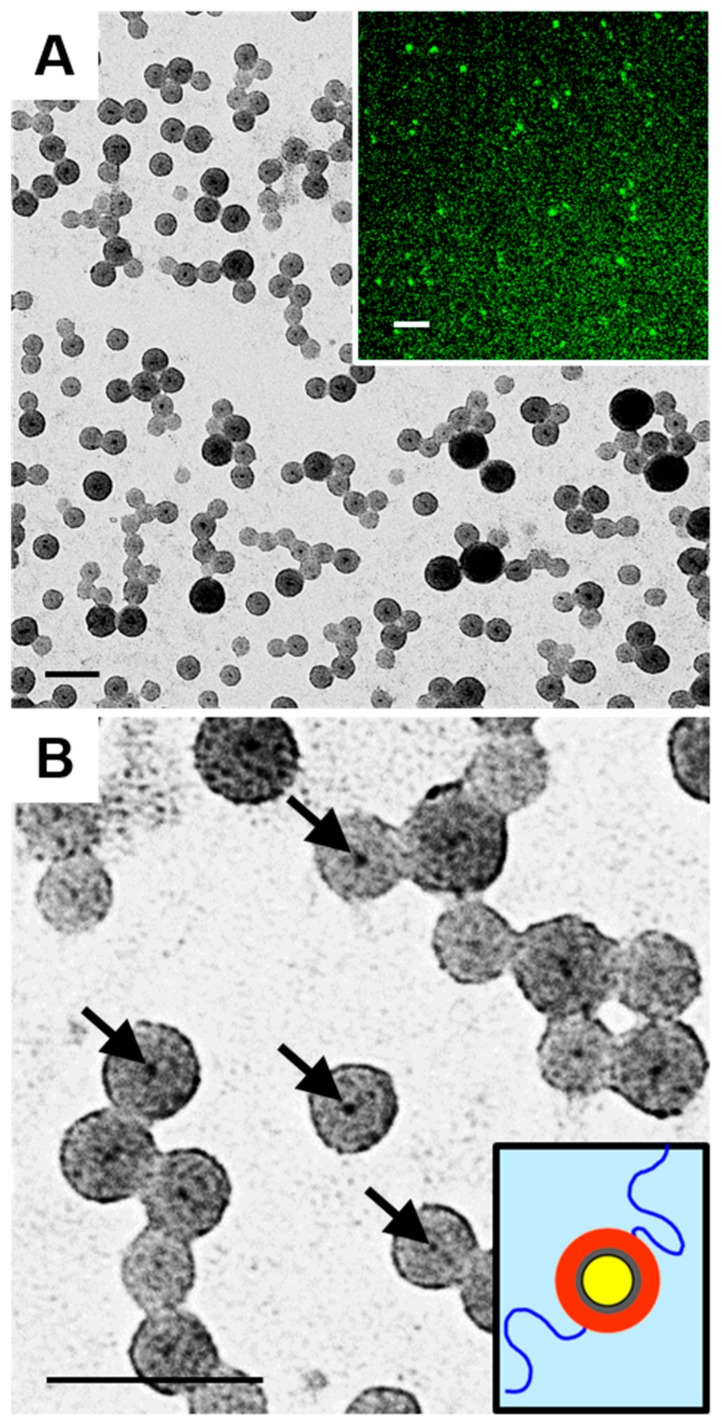
Core-shell QD-polymer nanoparticles formed from PS/PMAA-CdS in THF/water with *c*_0_ = 0.75 wt % and *R*_NaCl_ = 0 (no salt added). (**A**) Low-magnification TEM with LSCFM image (inset) showing QD emission. (**B**) High-magnification TEM with black arrows pointing to examples of QD cores with schematic of individual core-shell QD-polymer nanoparticle (inset). Scale bars are 100 nm, except in (A, inset) scale bar is 10 µm.

Figure 4 shows aqueous dispersions formed from PS/PMAA-CdS at an initial nanoparticle concentration of *c*_0_ = 0.75 wt % without the addition of salt before dropwise water addition (*R*_NaCl_ = 0). From low-magnification TEM images (Figure 4A), the resulting particles are exclusively spheres with a mean size diameter from TEM of 41 ± 1 nm. As clearly shown in high-magnification TEM images (Figure 4B), the vast majority of spheres possess a single dark dot in their center (black arrows point to examples), which is attributed to a CdS QD core surrounded by a condensed PS shell and stabilized by PMAA chains in water (inset, Figure 4B). The resulting core-shell nanoparticles are attributed to centrosymmetric intraparticle collapse of hydrophobic PS chains above a critical water content to form the uniform PS shell surrounding the central QD, with the hydrophilic PMAA chains protruding into the aqueous phase to maintain colloidal stability. Therefore, each core-shell particle is formed from a single PS/PMAA-CdS nanoparticle, suggesting that self-assembly is not favoured under the current conditions. The core-shell nanoparticles were found to be fluorescent due to the central cores of fluorescent QDs, as shown in the LSCFM image (Figure 4A, inset). Quantitative emission spectra should be similar to those of CdS QDs prepared within mixed brush micelles obtained previously by our group [16].

Further evidence that each core-shell nanoparticle consists of a single PS/PMAA-CdS unit is provided by comparing PS chain aggregation numbers from TEM images (Figure 4) with SLS results discussed previously (Table 1). Based on the mean diameter of core-shell spheres from TEM, we calculate the volume of the core-shell spheres:

$$V_{\mathrm{core}-\mathrm{shell}}=\frac{4}{3}\pi {\left(20.5\right)}^{3}=3.61\times 10^{4}{\;\mathrm{nm}}^{3}$$

As reported in a previous section, the mean radius of PS/PMMA-CdS cores that consists of a CdS QD and a surrounding layer of crosslinked PACd was determined to be *r*_core_ = 8.1 nm. Assuming the core of the core-shell particles to be the same, we calculate the core volume:

$$V_{\mathrm{core}}=\frac{4}{3}\pi {\left(8.1\right)}^{3}=2.24\times 10^{3}\;{\mathrm{nm}}^{3}$$

From these values, we obtain the volume of the PS shell:

$$V_{\mathrm{shell}}=V_{\mathrm{core}-\mathrm{shell}}-V_{\mathrm{core}}=36,100-2240=33,860\;{\mathrm{nm}}^{3}$$

which, assuming a bulk PS density of 1.04 g cm^−3^, yields the mass of a single PS shell:

$$m_{\mathrm{shell}}=1.04\;{g\;\mathrm{cm}}^{-3}\times 3.386\times 10^{-17}{\mathrm{cm}}^{-3}=3.521\times 10^{-17}g$$

We know the molar mass of an average 250-unit PS chain to be 26,038 g mol^−1^. Therefore, the mean number of PS chains in each shell will be:

$$Z_{\mathrm{PS}}=\frac{3.521\times 10^{-17}g}{26,038{\;g\;\mathrm{mol}}^{-1}}\times 6.02\times 10^{23}{\mathrm{chains}\;\mathrm{mol}}^{-1}=814\;\mathrm{chains}$$

By comparison, SLS data for PS/PMMA-CdS in THF gives a total chain aggregation number of *Z* = 850 ± 70 (Table 1), which, considering that 89% of the total chains are PS, gives *Z*_PS_ = 760 ± 60. Thus, the value for the core-shell particles from TEM (*Z*_PS_ = 814) agrees within error with the SLS value of individual PS/PMMA-CdS particles dispersed in THF. Since GPC results before and after hydrolysis suggest that aggregation numbers for PS/PMMA-CdS and PS/PMAA-CdS should be the same, this agreement between SLS and TEM results supports the conclusion that each core-shell particle (Figure 4) arises from intraparticle chain rearrangements of a single PS/PMAA-CdS unit upon water addition.

**Figure 5:**
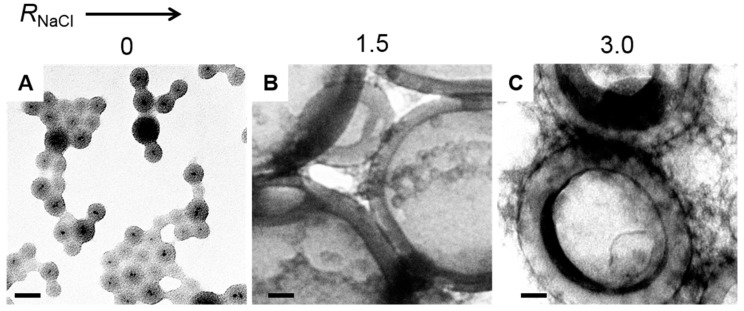
Effect of added salt content on self-assembly of PS/PMAA-CdS nanoparticles in THF/water mixtures. For all three preparations, *c*_0_ = 0.25 wt %: (**A**) *R*_NaCl_ = 0 (no salt added), (**B**) *R*_NaCl_ = 1.5, and (C) *R*_NaCl_ = 3.0. All scale bars are 50 nm.

We have previously shown that ABNPs exhibit salt-dependent self-assembly characteristics due to the charging of PAA chains in water [17]. Therefore, we investigated the effect of adding salt on the behaviour of PS/PMAA-CdS upon dropwise water addition. We found that for initial nanoparticle concentrations *c*_0_ > 0.25 wt %, macroscopic precipitation was observed at high salt content (*R*_NaCl_ = 3.0). Therefore, to compare the effect of salt content on the resulting morphologies, we held the initial nanoparticle concentration constant at *c*_0_ = 0.25 wt % and investigated the resulting morphologies formed for three salt contents: *R*_NaCl_ = 0 (no salt), *R*_NaCl_ = 1.5, or *R*_NaCl_ = 3.0 (Figure 5). From the resulting TEM images, we find that core-shell nanoparticles with a mean size of 37 ± 1 nm are formed in the absence of salt (Figure 5A) and are similar to those formed at the higher initial nanoparticle (Figure 4). The similarity in the sizes of core-shell particles formed at different initial nanoparticle concentrations further supports that these particles do not arise from self-assembly of multiple nanoparticles but rather form from intraparticle conformational rearrangements of individual nanoparticles. In contrast, water addition in the presence of salt at a ratio of *R*_NaCl_ = 1.5 is found to form vesicles with electron-dense bilayer interfaces, suggesting the self-assembly and interfacial packing of multiple QDs within each vesicle (Figure 5B). The mean thickness of the vesicle walls was determined from TEM images to be *t* = 30 ± 3 nm. When the salt ratio was increased to *R*_NaCl_ = 3.0, vesicles were also formed (Figure 5C), although with slightly thicker walls (*t* = 35 ± 2 nm) than those formed at the lower salt ratio.

**Figure 6:**
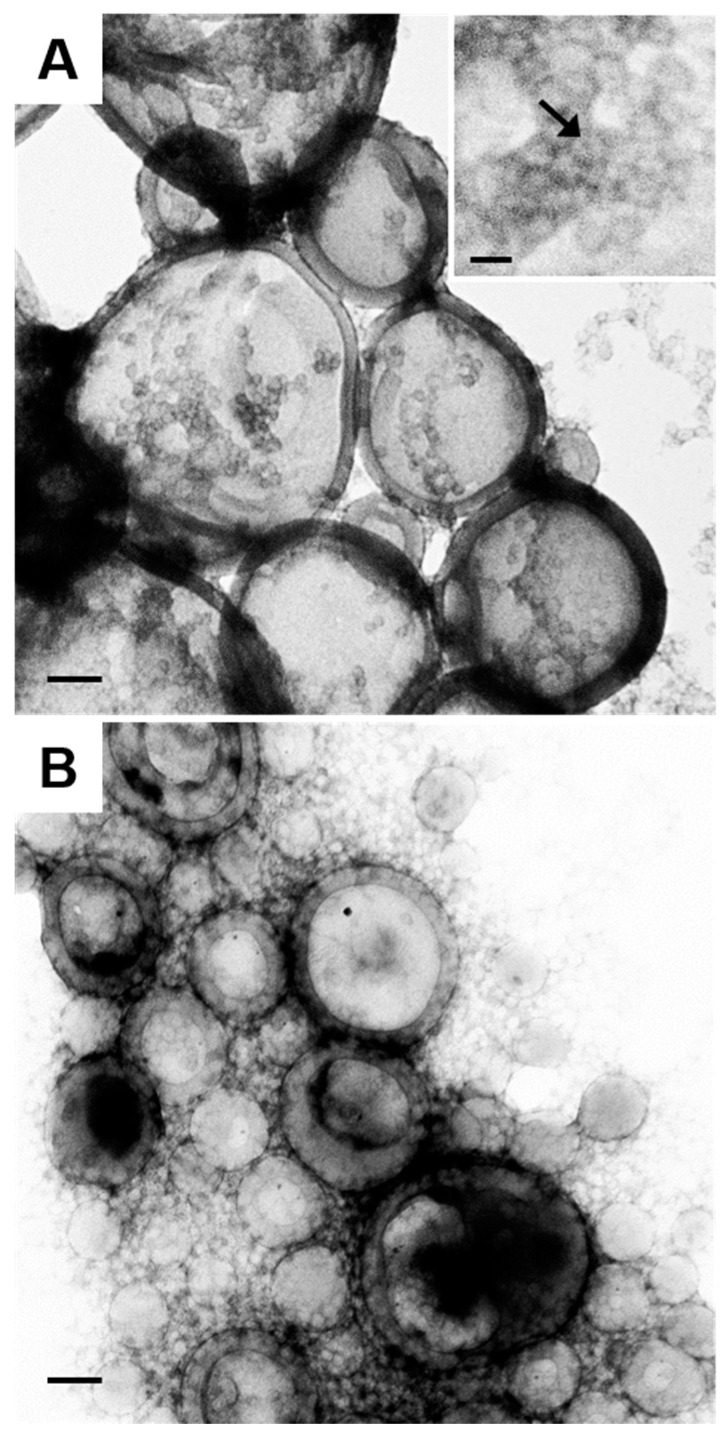
Low-magnification TEM images of QD-polymer vesicles formed from PS/PMAA-CdS in THF/water with *c*_0_ = 0.25 wt % and (**A**) *R*_NaCl_ = 1.5 and (**B**) *R*_NaCl_ = 3.0. Inset to (**A**) shows core-shell nanoparticles that coexist with vesicles. All scale bars are 100 nm.

Figure 6 shows lower-magnification images of vesicles formed at both salt ratios. Both TEM images in Figure 6 show that multiple small spherical particles coexist with the vesicles; close inspection reveals that these small particles appear to be individual core-shell nanoparticles (Figure 6A, inset). These core-shell nanoparticles may have formed from outliers in the distribution of PS/PMAA-CdS nanoparticles, or else they represent kinetically trapped unimers that were excluded from vesicle formation by vitrification of the PS phase.

**Figure 7:**
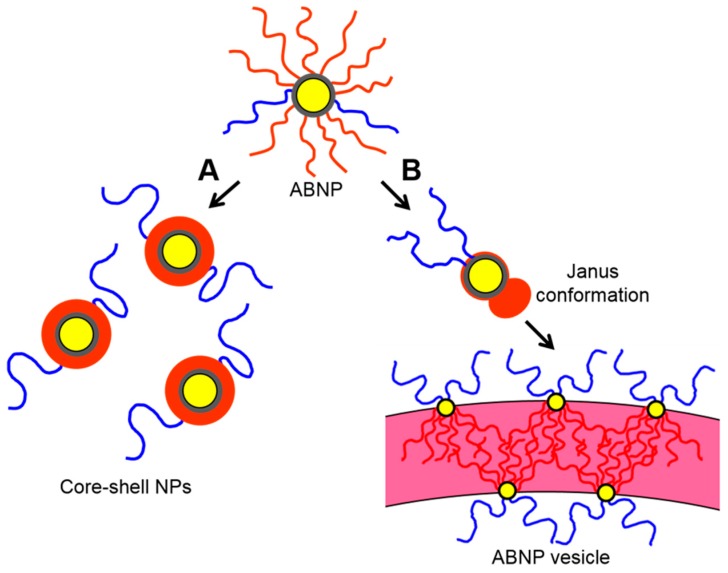
Schematic showing two conformational rearrangement pathways for PS/PMAA-CdS mixed brushes above the critical water content. (**A**) Centrosymmetric condensation of PS with no salt addition and (**B**) non-centrosymmetric condensation of PS with salt addition and subsequent self-assembly of Janus particles into QD-polymer vesicles.

Figure 7 shows two pathways for intraparticle conformational rearrangements above the critical water content, with A and B leading to individual core-shell nanoparticles and nanoparticle self-assembly into vesicles, respectively. Both pathways involve hydrophobic and/or hydrophilic chains that wrap around the core to adopt the most energetically favourable conformation, which is enabled by the large radius of gyration of the brush (*r*_g_ = 123 nm) compared to the small radius of the core (*r*_core_ = 8 nm). Similar conformational rearrangements for mixed brushes above a critical water content have been previously attributed to large size discrepancies between long polymer chains and small inorganic cores [6,15,17]. In path A, the PS blocks condense on the core in a centrosymmetric manner, forming a uniform PS shell. The PMAA chains will be significantly charged at neutral pH and so will rearrange to maximize interchain distances while providing colloidal stability to the hydrophobic shell. Path A will be favourable under conditions in which electrostatic repulsive interactions between PMAA chains are dominant, explaining the formation of core-shell nanoparticles at low ionic strength (i.e., in the absence of added salt). In path B, the PS blocks condense on the core in a non-centrosymmetric manner, forming a Janus structure in which PS and PMMA chains segregate to opposite faces of the nanoparticle. The resulting symmetry breaking leads to anisotropic interactions between nanoparticles and self-assembly into vesicle bilayers with a distinct PS/PMAA interface. Since path B requires close intraparticle packing of PMAA chains during chain segregation; this path will be favourable under conditions in which electrostatic repulsive interactions between PMAA chains are less important due to screening effects (i.e., at high ionic strength). This explains why self-assembly and vesicle formation is only observed in the presence of salt.

The salt-dependent tunability between discrete core-shell nanoparticles and complex hierarchical QD-polymer vesicles is an interesting and unique feature of this amphiphilic QD-polymer system. This behaviour may be partly attributed to the high packing density of chains in the mixed brush (~1 chain/nm^2^), which leads to strong electrostatic contributions to intraparticle conformational rearrangements as discussed above in connection with Figure 7. In contrast, our previously reported system of PS/PMMA-CdS nanoparticles underwent self-assembly both in the presence and absence of salt, although the specific self-assembled morphologies were salt-dependent [17]. In that case, the packing density of chains in the mixed brush was lower (~0.77 chain/nm^2^) [16] such that electrostatic contributions to intraparticle rearrangements were consequentially weaker. Another distinct feature of the current ABNPs is their highly asymmetric brush composition. The high hydrophobic chain content should allow the formation of continuous PS shells with a lower entropic penalty than for more symmetric compositions, further contributing to the favourable formation of core-shell nanoparticles in the current system. 

## 4. Conclusions

Using a mixed micelle approach, we produced amphiphilic brush nanoparticles (ABNPs) that consisted of CdS QD cores with surface layers of densely grafted (σ = ~1 chain/nm^2^) and asymmetric (*f*_PS_ = 0.9) mixed polymer brushes that contained hydrophobic PS and hydrophilic PMAA chains (PS/PMAA-CdS). Following rigorous characterization of the ABNPs and their precursors, we investigated the morphologies formed by PS/PMAA-CdS in THF/water mixtures (after dialysis to remove residual organic solvent). In aqueous media, the PMAA chains were significantly charged, such that conformational rearrangements during dropwise water addition and subsequent morphologies were found to depend strongly on prior salt addition. As a result, the addition of water to THF dispersions triggered one of two pathways for the conformational rearrangement of the mixed brush. (A) In the absence of salt, centrosymmetric condensation of PS chains formed individual core-shell QD-polymer colloids. (B) In the presence of salt, non-centrosymmetric condensation of PS chains formed Janus particles, with anisotropic interactions leading to amphiphilic self-assembly into QD-polymer vesicles. To our knowledge, this is the first example of an ABNP building block that can form either discrete core-shell colloids or self-assembled superstructures depending on simple changes to the chemical conditions (i.e., salt addition). Such finely tuned and dramatic morphological control in hierarchical QD-polymer materials could inform numerous future applications, including biolabeling, sensing, photonics, and nanomedicine. 

## Figures and Tables

**Table 1:** Structural Characteristics of PS/PMMA–CdS from SLS and DLS in THF.

*f*_PS_	*Z*	*r*_g_ nm	*σ*, chains nm^−2^	*r*_h,0_ nm	*t*_b_, nm	*extension,* %	*r*_g_/*r*_h,0_
0.89	850 ± 70	123 ± 9	1.02 ± 0.08	29.0 ± 0.2	21 ± 1	37 ± 1	4.2 ± 0.1

## Supplementary Materials

The supplementary materials are available online at http://www.mdpi.com/2073-4360/10/3/327/s1.
