# Supplementary material for: Amphiphilic Quantum Dots with Asymmetric, Mixed Polymer Brush Layers: From Single Core-Shell Nanoparticles to Salt-Induced Vesicle Formation
Source: Polymers (Basel). 2018 Mar 16;10(3):327. doi: 10.3390/polym10030327 (PMC6414905; doi:10.3390/polym10030327)
Supplement: Supplementary file 1 [file polymers-10-00327-s001.pdf]

Amphiphilic Quantum Dots with Asymmetric Mixed Polymer Brush Layers: from Single Core-Shell Nanoparticles to Salt-Induced Vesicle Formation

Brian R. Coleman and Matthew G. Moffitt*

Department of Chemistry, University of Victoria, P.O. Box 3065, Victoria, BC, Canada V8W 3V6

Supporting Information

Additional data on the synthesis and characterization of PS/PMAA-CdS nanoparticles and their precursors (as described in the main text) are available.

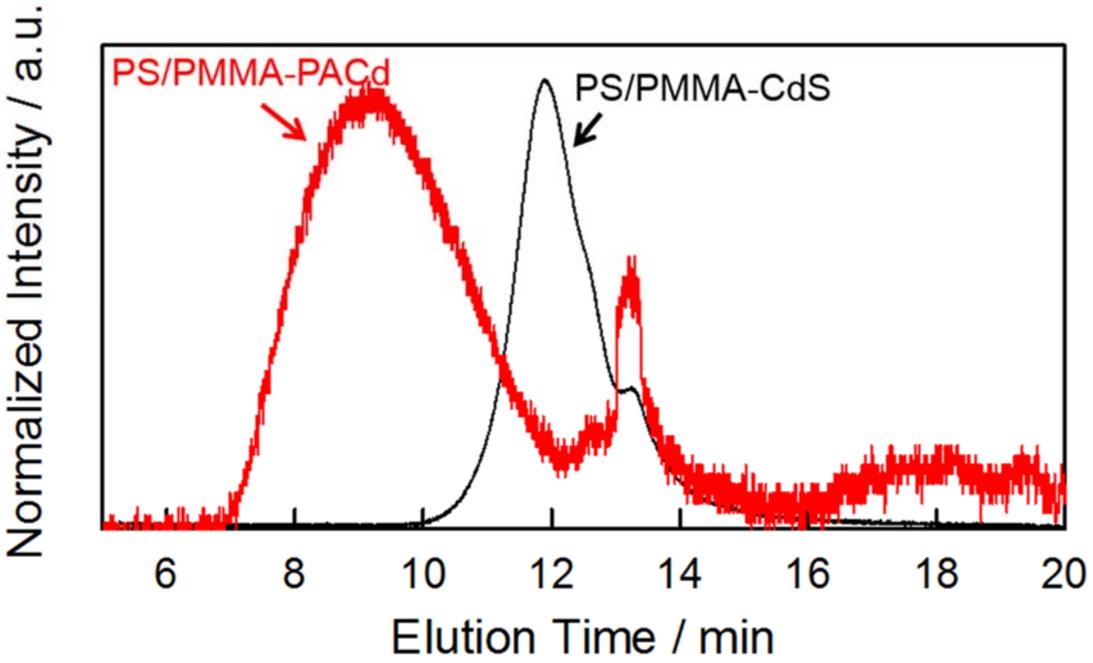

Figure S1. GPC chromatograms comparing PS/PMMA-PACd mixed micelles (red line) with hydrophobic mixed brush nanoparticles PS/PMMA-CdS (black line).

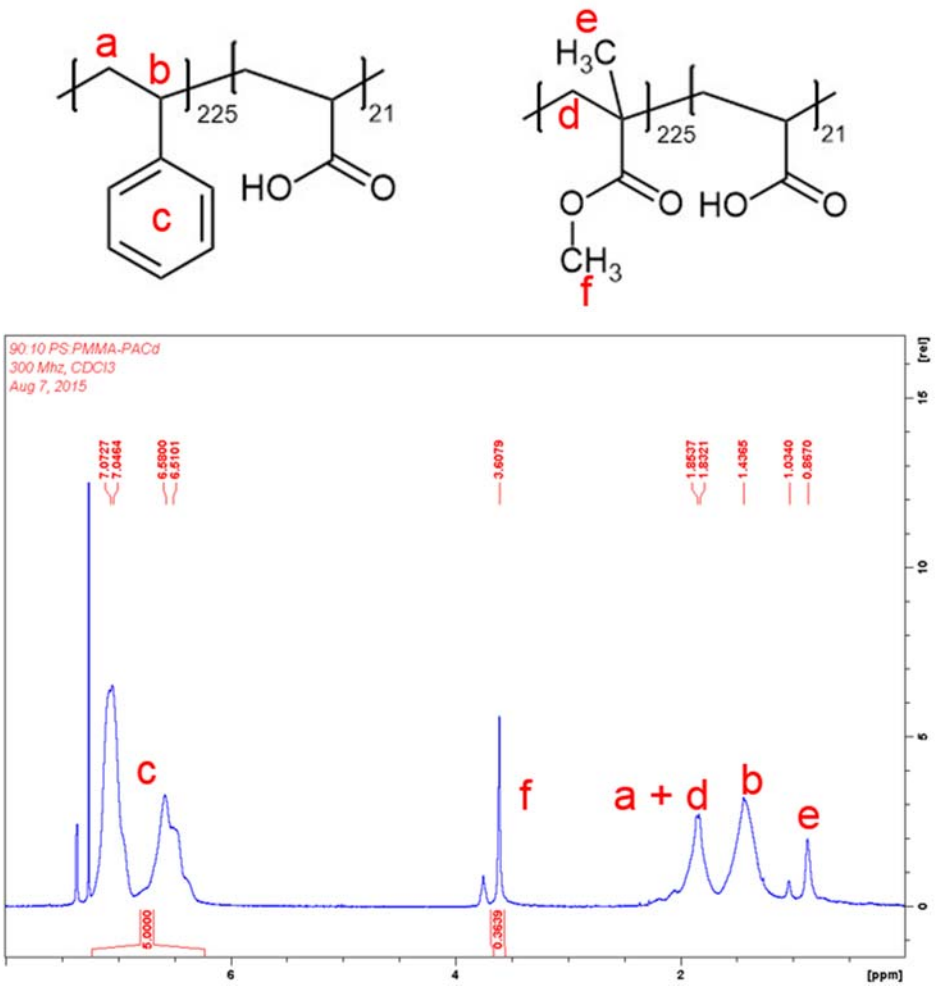

Figure S2. Structure of block copolymer components with proton designations and ^{1}H NMR data and peak assignments for PS/PMMA-PACd inverse micelles. The mole fraction of PS chains relative to the total number of PS and PMMA chains was determined from relative peak integrations of the aromatic protons of PS (6.2-7.2 ppm) and the methyl ester protons (3.6 ppm) of PMMA.

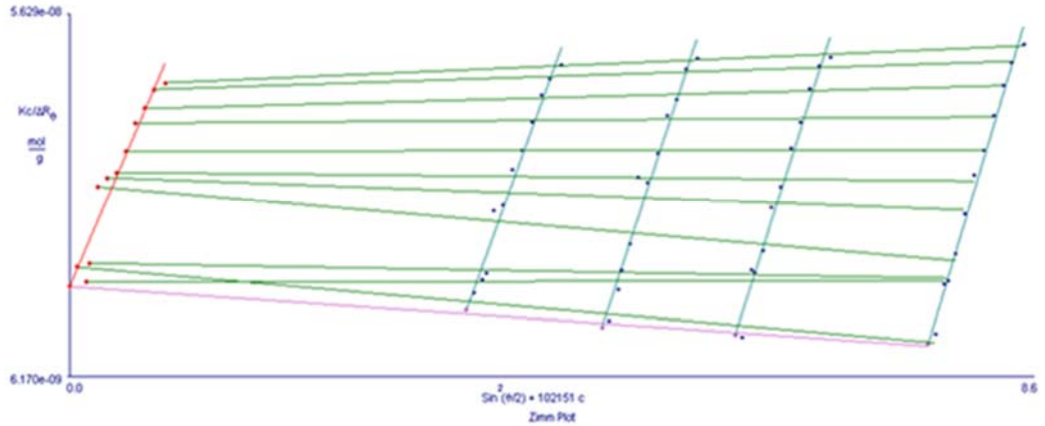

Figure S3. Zimm plot from SLS data for PS/PMMA-CdS. SLS experiments were performed using THF as a solvent.

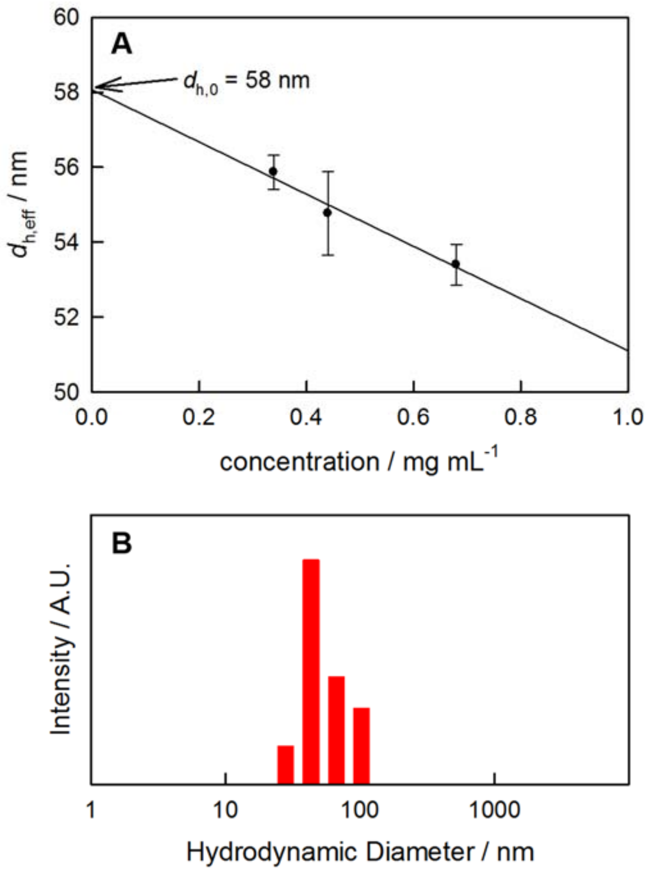

Figure S4. (A) Plot of effective hydrodynamic diameters, $d_{h,eff}$, versus concentration from cumulant analysis of DLS data for PS/PMMA-CdS in THF, showing extrapolation of linear regression line to determine $d_{h,0}$. (B) CONTIN size distribution for PS/PMMA-CdS in THF.

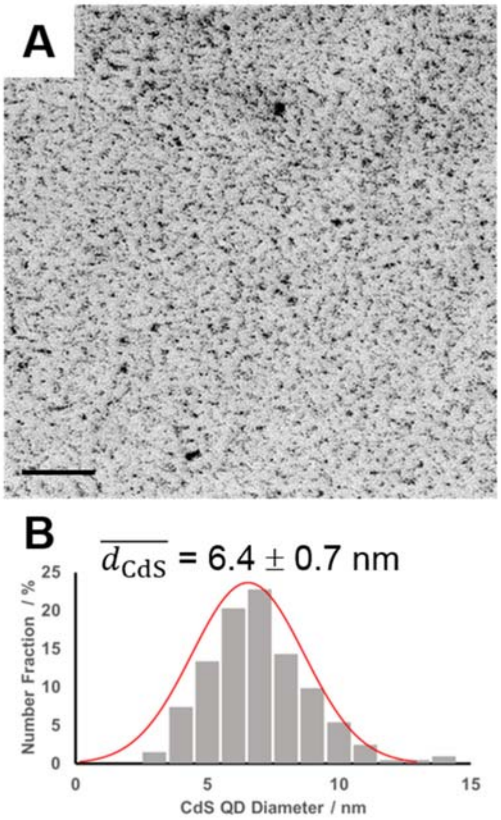

Figure S5. (A) TEM showing dark CdS QD cores and (B) associated size distributions of benzene-cast PS/PMMA-CdS. Scale bar is 100 nm.

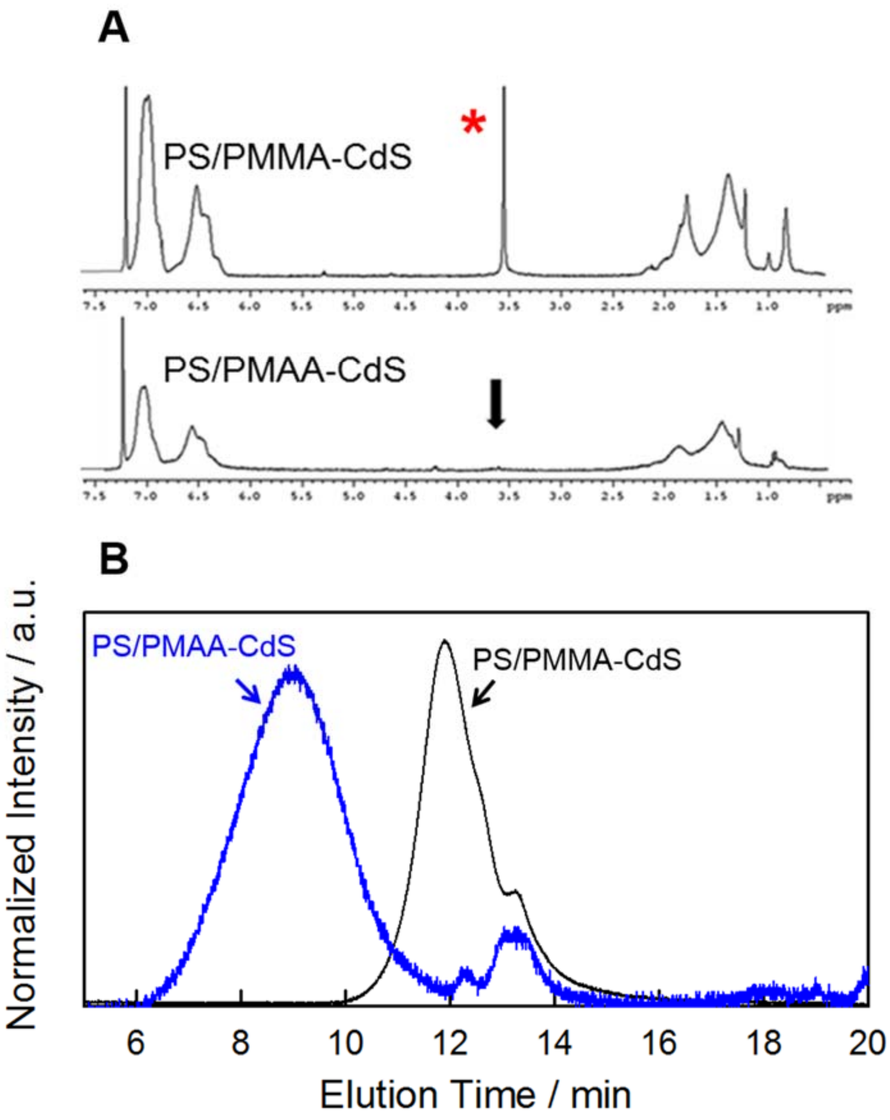

Figure S6. (A) ^{1}H NMR data before and after hydrolysis of PS/PMMA-CdS to PS/PMAA-CdS showing disappearance of methyl ester peak (red asterisks) indicating complete hydrolysis. (B) GPC chromatograms comparing hydrophobic PS/PMMA-CdS (black line) with amphiphilic PS/PMAA-CdS (blue line). Chromatograms are shifted for display purpose using single chain peaks as reference points. The chromatograms show that single chain peak intensities did not increase during conversion of PS/PMMA-CdS to PS/PMAA-CdS, indicating that the mixed brushes retained their structural integrity during the hydrolysis step.

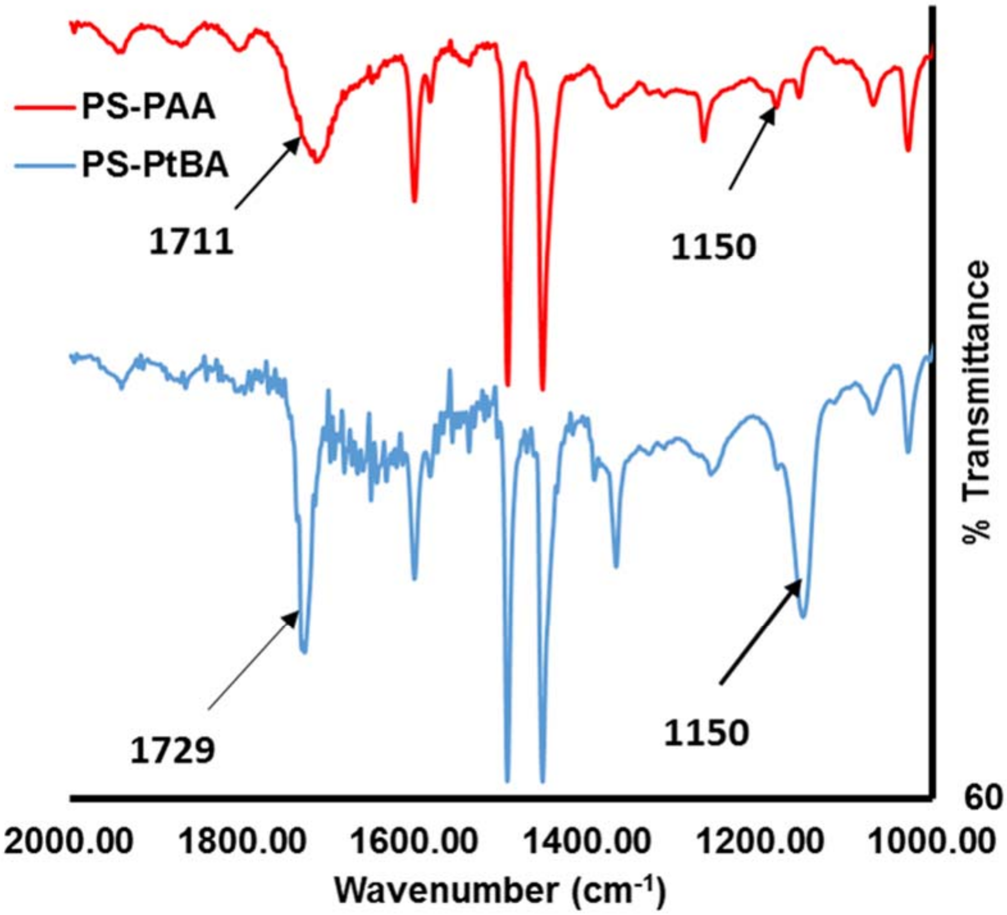

Figure S7. FTIR of the PS-*b*-PAA mixed micelle component and PS-*b*-PtBA starting material. Complete hydrolysis of the P*t*BA block to PAA was indicated by disappearance of the C-O stretch peak at 1150 cm^{-1} and broadening of the original carboxylate peak at 1729 cm^{-1}.
